# A Systematic Review on the Association Between Bilingualism and Theory of Mind in Adulthood

**DOI:** 10.3390/bs15060815

**Published:** 2025-06-13

**Authors:** Rowena J. Xia, Brian W. Haas

**Affiliations:** 1 Department of Psychology, Concordia College, Moorhead, MN 56562, USA; 2 Department of Psychology, University of Georgia, Athens, GA 30602, USA; bhaas@uga.edu

**Keywords:** bilingualism, theory of mind, adulthood

## Abstract

Previous research on the relationship of bilingualism and theory of mind has largely focused on children. However, several recent studies of the theory of mind have found differences in theory of mind processing among older populations, namely adults. Given that language has been found to play an important role in the successful theory of the mind task performance of adults, it is valuable to understand the relationship of the language ability of bilingualism and theory of mind in adults. The specific focus is on studies comparing monolinguals and bilinguals in a theory of mind assessment for an adult sample. In this systematic review, we reviewed and analyzed these studies and conducted a meta-analysis. Among the studies included for meta-analysis (k = 7), we found a significant small-to-medium effect size (*d* = 0.402, *p* < 0.0001), indicating a bilingual advantage among adults. A variety of different measures for theory of mind were included in these studies. More studies are required to better understand the relationship between multiple language processing and social cognition among adults to better understand this gap in the literature.

## 1. Introduction

The question of whether there is a bilingual advantage, in which bilinguals have an advantage over monolinguals in terms of improved abilities on various social and cognitive tasks, is a continuously asked question in the field of bilingualism. A growing body of research (Nichols et al., 2020; Paap et al., 2015; Schroeder, 2018; Ware et al., 2020) has focused on understanding whether this advantage exists, especially as it relates to the theory of mind (ToM). ToM, as conceived as the ability to understand the mental state of others, has been assessed in many ways but all are rooted in some understanding of taking the perspective of others, such as through a false belief test like the classic Sally–Anne unexpected-transfer test (Baron-Cohen et al., 1985). There has been a growing push to recontextualize ToM in models that better reflect the complexity of ToM, such as understanding it as having lower and higher levels as well as incorporation of both cognitive and social components (Apperly, 2012; Schaafsma et al., 2015; Schurz et al., 2021). These perspectives advocate for a more complex understanding of what ToM is, which also includes how ToM is not just understanding something like the Sally–Anne task at a young age, but instead evolving and growing as you age, and is best understood with an array of different tasks (Apperly, 2012). Of course, ToM is considered an important step in the cognitive development process in childhood, which comes to exist around age 4 (Wellman et al., 2001). Developing this ability is important for a child as a milestone as it indicates an important level of social-cognitive understanding: separating oneself and others’ perspectives. However, it is important to study adults to better understand how the development of ToM continues beyond childhood. Studying adults also helps with investigating the nuances of advanced, higher level ToM functioning in a mature, adult system, including individual differences that exist, as well as to understand how ToM abilities interact with other social-cognitive functions for adults (Apperly et al., 2009).

As many studies have indicated, there are differences in ToM capabilities found amongst both child and adult populations (Apperly et al., 2009; Epley et al., 2004; Qureshi et al., 2020). A systematic review of studies on bilingualism and ToM for children found a significant difference between bilinguals and monolinguals that indicated a small bilingual advantage (*d* = 0.22), which became a medium bilingual advantage (*d* = 0.58) when the ToM scores were corrected for the proposed bilingual disadvantage of having lower receptive language proficiency than monolinguals (Schroeder, 2018). This meta-analysis supports a ToM advantage in children that may demonstrate just a small effect but which also shows that other factors, in this case language proficiency differences, may contribute to a greater bilingual advantage. Explanations for this advantage have highlighted factors like enhanced executive control and metalinguistic understanding (Goetz, 2003; Buac & Kaushanskaya, 2020). It has specifically been found that the language-switching experience that bilinguals navigate by nature of needing to switch between language systems is key to bilinguals being better at cognitive control processes of executive functions, including processes that are involved in tasks of ToM (Verreyt et al., 2016). The development of these ToM abilities has been found to continue well beyond childhood, including late adolescence (Dumontheil et al., 2010). To study this continuously developing ability into adulthood, studies conducted with adults have taken different approaches such as using eye-tracking to address the advanced capabilities of adults on ToM (Rubio-Fernández & Glucksberg, 2012), using more advanced ToM tasks (Navarro & Conway, 2021), and using self-report measures of empathy while also investigating the interaction of bilingualism and gender (Tarighat & Krott, 2021). These studies all found significant differences in adult bilinguals and monolinguals in varied ToM assessments, including an interaction of gender and bilingualism (Tarighat & Krott, 2021). This interaction found that bilingualism enhanced ToM in men in a way that balanced that enhanced ToM ability in women found with monolinguals, indicating the male-specific enhancing effect of bilingualism. The focus of this study will not be gender, but this finding provides important context that must be considered in future studies or studies that investigate the role of gender.

While many studies focus on neurodivergent adults, this review will focus specifically on neurotypical populations to understand ToM in neurotypical populations. Specifically, neurotypical adults are found to have individual differences in processing on ToM tasks (Apperly et al., 2009; Dumontheil et al., 2010). For adult populations, it has been found that egocentric biases exist when adults are tasked with taking the perspective of another in an online communication game (Keysar et al., 2003). In this task, participants playing a referential communication game with a figure deemed a “director” were tasked with moving objects around a grid in which some were only visible to the participant and not the “director”, and then were asked to interpret the perspective of the director (Keysar et al., 2000). Essentially, participants were asked to move specific objects on a grid based on instructions from the director figure on the screen, in which some conditions involved objects that were blocked from the view of said director. In those cases, the task was to pick the correct object to move based on the perspective of the director. The intention of this task is to assess how well a participant can distinguish between their perception and the perception of another, with in this case the other being the director of the task. While adults can reflect and correctly identify what the director falsely believes, they would also spontaneously make the incorrect choice or were delayed in identifying the intended object, indicating the inconsistent reliability of ToM reasoning in adults (Keysar et al., 2003).

In investigating the reasons for the limits of ToM reasoning in adults, particularly in terms of egocentric errors, it is suggested that adults can be inefficient in interpreting what others say (Apperly et al., 2009). Specifically, participants were found to be limited in ability to hold both the perspective of the director and use it to guide processing at the same time. Eye-tracking analysis reveals that there are distinct eye fixation patterns for taking the perspective of oneself compared to the perspective of others in visual perspective-taking (Ferguson et al., 2017). There was stronger bias toward focusing gaze to the location of the other in the other perspective-taking condition compared to focusing on multiple possible locations in the self-perspective condition (Ferguson et al., 2017). These results help explain some of the attentional and cognitive mechanisms underlying why adults still have biases of ToM.

In an experiment on adults and their completion of a nonverbal ToM task while completing a verbal task, it was found that language played an interfering role and disrupted the ability of adults to complete the ToM task (Newton & de Villiers, 2007). This would point toward a role of language in the disruption of adults on ToM reasoning, given the deteriorating performance on ToM when language capacity is occupied by another task. This relationship of language with ToM also plays a role in supporting a potential important factor of bilingualism, another feature of language capacity. In fact, the study of adults is specifically argued to be important for understanding the role of language in ToM because it is only in adults in which language has fully developed and matured and can thus be studied to its fullest capacity (Apperly et al., 2009).

It is also important to address the studies that have indicated a lack of evidence supporting individual differences for ToM in neurotypical adults, including findings that ceiling effects are common (Yeung et al., 2024). This review indicates that it is important to move away from the measures that show ceiling effects and move toward developing new measures, especially ones that consider age and culture (Yeung et al., 2024). Thus, it is argued that ToM in adults should continue to be studied, but perhaps using different or new measures. This review thus also serves the purpose of obtaining a picture of what existing measures have been used to help inform the direction of future research.

Despite these limitations, given the research on differences of adults in their ToM abilities, this would indicate that there should similarly be more research on the relationship of bilingualism with ToMin adults and not just research with children. While there have been several reviews and meta-analyses about the relationship of bilingualism and ToM (Schroeder, 2018; Feng et al., 2023), there have been none to focus entirely on studies of adult populations. This gap in the literature was noted by Feng and colleagues in their review (Feng et al., 2023). The purpose of this study is to conduct a systematic review on the empirical research that exists on the relationship of bilingualism and ToM specifically with adult populations to answer the questions about the nature of the bilingual relationship with ToM for adults. The systematic review will focus on studies that compare bilingual and monolingual samples for a measure of ToM in which the sample comprises adults 18 years of age and older. Additional studies will be discussed that do not meet the inclusion and exclusion criteria that are relevant to reviewing the research literature on adults, bilingualism, and ToM. It is predicted that a significant small effect will be found across the studies reviewed in the systematic review, indicating a small bilingual advantage.

## 2. Methods

The inclusion and exclusion criteria are included in Table 1. These criteria emphasize the kinds of study designs of focus for this systematic review and the definitions utilized for the key factors of bilingualism, ToM, and neurotypical adults. For the purposes of this review, bilingualism is conceptualized as speaking two or more languages as self-reported by participants. For the purposes of this review, no criteria for proficiency of use, frequency of use, or age of exposure were included in defining bilingualism, other than what the studies themselves already utilized. The review only focuses on any studies that include people who self-reported in some way as being bilingual. Studies that only focused on bilinguals were not included since the intention for this review is to examine the potential bilingual advantage, which requires a monolingual comparison group. The range of ToM tasks included was cast as a wide net to examine the array of different components of ToM reasoning. However, neurological studies were not included as the focus for analysis was more on any measures that involve behavioral, cognitive, or self-report data on the capacity of ToM and not neural pathways or locations.

**Figure 1:**
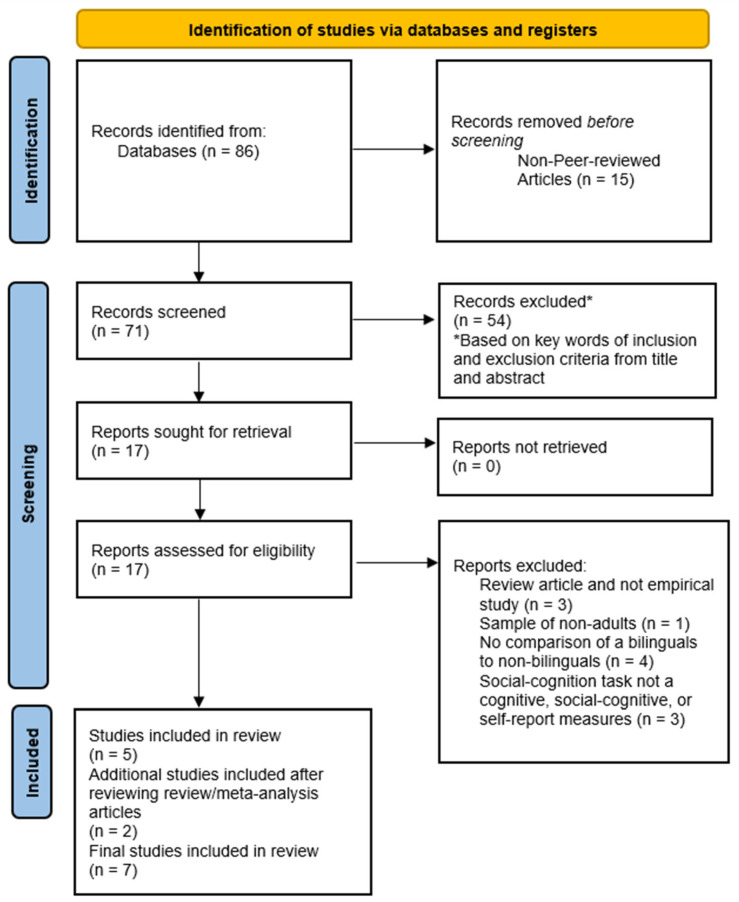
PRISMA Diagram.

PRISMA guidelines were followed with the PRISMA diagram illustrating the process in Figure 1. A literature search was conducted for the databases of PSYCArticles and PSYCInfo on 7 February 2025 and found 86 articles, screened by the first author and using no automation tools. The second author served as a secondary reviewer for the screening process and worked independently. There were no publication criteria in terms of dates of publication or types of journals. After excluding any non-peer-reviewed articles, 71 articles remained. The search terms used were the following: bilingual* OR multilingual* OR trilingual OR “dual language*” OR “two languages” OR “second language” OR “heritage language*” AND “social cognit*” OR “theory of mind” OR ToM OR TOM OR false belief* OR “false-belief*” OR FB OR mentalizing* OR mentalising* OR “perspective taking*” OR perspective-taking* AND adult*. Following initial screening of the titles and abstracts of the 71 articles that met the inclusion criteria, 17 articles were included for further review. This screening process for the abstracts involved looking for any keywords that matched the three inclusion criteria being met based on the information provided in the abstract. Only abstracts that had keywords that matched all three of the criteria were included for subsequent evaluation. Any lack of clarity of one of the inclusion criteria meant that the article would be included for subsequent evaluation. Among these 17 articles, 3 articles were excluded after further screening for being review articles and not empirical articles, 1 was excluded for having a sample of non-adults, 4 were excluded for lacking a comparison of bilinguals and non-bilinguals, and 3 were removed for not including a social cognitive task on ToM (e.g., lexical and semantic analysis, neurological data). Subsequently, 5 articles remained that met these inclusion and exclusion criteria. For the next step, a scoping review of tasks and study designs on assessing ToM in bilinguals was carried out and two additional studies were found and included for analysis (Feng et al., 2023). These processes ended up bringing the total number of studies included for analysis to N = 7.

Data were collected for those 7 studies based on the screening criteria by the first author from each report using what was available in the report itself and from any additional data published online. The primary results that compared ToM capacity of monolinguals and bilinguals were the focus of which data to utilize. Cohen’s *d* was used as a standardized measure for a mean difference effect size comparing monolinguals and bilinguals across studies (Lakens, 2013). Reported Cohen’s *d* values were used (Chung-Fat-Yim et al., 2022; Navarro & Conway, 2021). One study calculated the effect size based on data provided by the authors (Tarighat & Krott, 2021). In other cases, Cohen’s *d* was calculated based on provided statistics of means, variances, and sample sizes, and other statistics provided (Cox et al., 2016; Ikizer & Ramírez-Esparza, 2018; Rubio-Fernández & Glucksberg, 2012; Ryskin et al., 2014). The type of measure used, the number of participants for the monolingual and bilingual groups, the average of the participants, and the *p*-value for determining whether a bilingual advantage was found were the other information collected. The assessment of risk of bias was completed by the first author and confirmed independently by the second author. Any potential publication bias was investigated using a funnel plot to test any asymmetry. Additionally, a forest plot was generated to illustrate the effect size distribution for this set of studies included in the meta-analysis and review. The meta-analysis method utilized was the random-effects model analysis, which required effect sizes and standard errors of each study using R and the meta package (Balduzzi et al., 2019). The confidence intervals reported indicated the level of certainty in the results found. The metagen function was also used to conduct a test of heterogeneity for the pooled effect size found. Given the small sample size for studies included, as well as the small sample for each ToM measure used, no further subgroup analysis for causes of heterogeneity or sensitivity analyses were conducted, although the heterogeneity of the measures of ToM used will be discussed as important context of these results. No additional assessments for the risk of bias were included.

The overview systematic review protocol used was registered and can be found on the Open Science Framework registries, with no additional amendments: https://osf.io/nx6h3, accessed on 13 May 2025. A specific review protocol was not prepared outside of the general guidelines.

## 3. Results

All analyses were conducted in R (R Version 4.3.2). Table 2 displays the studies included in the systematic review (N = 7), including the ToM measure that was used in the study, the sample sizes, the average ages, the *p*-values found, and the effect sizes. Four of the studies found a bilingual advantage in which the difference was statistically significant. Two studies found non-significant differences between the groups, which indicated the lack of a bilingual advantage (Chung-Fat-Yim et al., 2022; Cox et al., 2016). One study found that there was no significant difference, and in fact bilinguals actually made more errors according to the visuospatial ToM measure (Ryskin et al., 2014). Among the four studies indicating a bilingual advantage, two of them found a medium effect size (Navarro & Conway, 2021; Rubio-Fernández & Glucksberg, 2012), while the other two studies found a small-to-medium effect size (Ikizer & Ramírez-Esparza, 2018; Tarighat & Krott, 2021).

**Figure 2:**
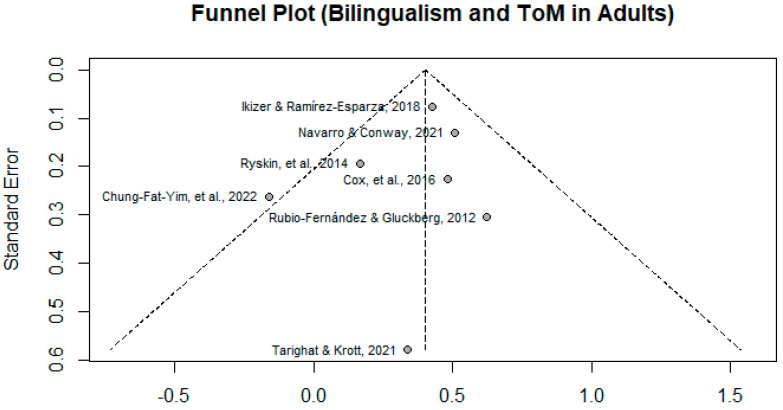
Funnel plot for meta-analysis of studies. (Ikizer & Ramírez-Esparza, 2018; Navarro & Conway, 2021; Ryskin et al., 2014; Cox et al., 2016; Rubio-Fernández & Glucksberg, 2012; Tarighat & Krott, 2021; Chung-Fat-Yim et al., 2022).

**Figure 3:**
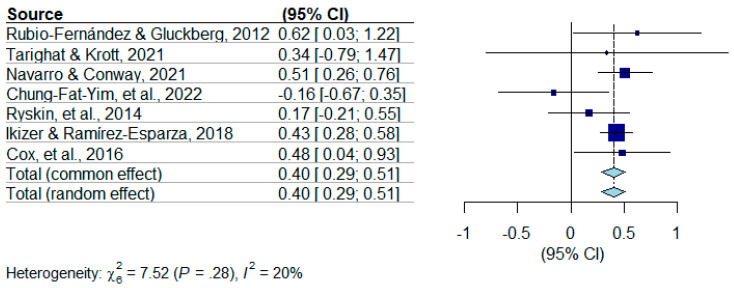
Forest plot for meta-analysis of studies. (Chung-Fat-Yim et al., 2022; Cox et al., 2016; Ikizer & Ramírez-Esparza, 2018; Navarro & Conway, 2021; Rubio-Fernández & Glucksberg, 2012; Ryskin et al., 2014; Tarighat & Krott, 2021).

A funnel plot (Figure 2) was generated to analyze potential publication bias. A visual inspection of the funnel plot does not appear to demonstrate asymmetry, indicating a lack of publication bias. Using the random-effects model analysis, the estimated effect was found to be *d* = 0.402, *p* < 0.0001, 95% CI [0.2901, 0.5143], indicating a significant medium effect size. The results indicate a bilingual advantage found in adults. The tests of heterogeneity for the model indicate that it is unlikely that there is heterogeneity among the studies with τ^2^ = 0, CI [0.0000, 0.2727], and *Ι*^2^ = 20.2%. Cochran’s Q test statistics indicate a *p*-value of 0.276, which indicates a non-significant test of heterogeneity for this model. Given the small number of studies included (N = 7), it is with caution that these tests of heterogeneity are interpreted, especially given that every study included a different type of ToM measure. The sensitivity and robustness of this meta-analysis is heavily restricted by the sample size (N = 7). The forest plot for the studies included in this meta-analysis can be found in Figure 3.

Data extracted, data used for analyses, R script code, and other materials used in this review can be provided upon request.

## 4. Discussion

The current body of research on bilingualism and ToM is found to be very limited in quantity, but indicates a bilingual advantage with a small-to-medium effect size. Given the limited sample size of studies, it is important to exercise caution in drawing any sweeping conclusions about these results. Nonetheless, this significant small-to-medium effect size points to a need for more studies conducted to understand the role of bilingualism in ToM for an adult population. Given the variety of measures used for assessing different aspects of ToM, it would appear to be useful to continue to study bilingualism and ToM in adults to better understand this relationship, including continued use of a variety of assessments for ToM. It would also be helpful to gain insight into the role of language, which is much more weakly understood for children who have more limited development of language.

As can be seen through the forest plot, the majority of the seven studies have a 95% confidence interval indicating a significant effect (Figure 3). Several studies have an effect size settling close to the total random effect, *d* = 0.402. Additionally, the test of heterogeneity of these studies indicates that the inconsistency is very low, *I*^2^, which supports a tentatively confident interpretation of this meta-analytic result as robust, with the caveat of the limited sample size (n = 7).

Reviewing the studies more in depth, there is a variety of approaches taken to measure ToM. One study takes the traditional Sally–Anne task used for children and uses eye-tracking to assess a different aspect of ToM performance for adults outside of accuracy (Rubio-Fernández & Glucksberg, 2012). This study was able to find a significant difference, but to our knowledge, no other studies have looked at ToM in adults through the Sally–Anne Task and with eye-tracking. Whether this approach should be repeated in the future can be explored in future studies, including whether eye-tracking could be utilized with other ToM scenarios outside of the classic Sally–Anne version. One study used the Director Task (Dumontheil et al., 2010), which is a ToM measure specifically used with adults and focuses on following a target item from the perspective of a director as it is moved around a 4 × 4 shelf (Navarro & Conway, 2021). This task contains more complexity in terms of movement of the object than the more traditional tasks like the Sally-Anne task, which allows it to be a better task to assess adult comprehension of ToM. There is more processing needed to understand the Director Task than the Sally–Anne Task, even though both have similar underlying mechanisms that another person is not able to see something. Perhaps the Director Task should be utilized more, especially given the significant medium effect size found (*d* = 0.510). Ryskin and colleagues instead focused on a visuo-spatial perspective task and focused on the number of errors committed in navigating directions with objects, with similarities to the Director Task (Ryskin et al., 2014). This is similar to previous studies of ToM in adults that utilized eye tracking to understand attentional patterns related to the completion of these tasks (Ferguson et al., 2017). However, it is notable that this study found no significant difference and did not support the bilingual advantage. This would mean that perhaps visuospatial tasks are not the direction in which future studies should go for assessing ToM in adults. As one of the most literal assessments of ToM, visuospatial tasks rely on visual understandings and thus are most likely to reach ceiling effects for adults who would most definitively be past this point of development, compared to more abstract ToM reasoning.

Tarighat and Krott explored both the effect of bilingualism as well as gender differences in terms of ToM, and notably found interactional effects between the two variables, with regard to the self-reported perspective-taking assessment completed in the study (Tarighat & Krott, 2021). This assessment is a subscale of the Interpersonal Reactivity Index (IRI), which seeks to assess self-reported individual differences in empathy (Davis, 1980). The perspective-taking subscale of the IRI focuses on the tendency for being able to spontaneously take the point of view of someone else psychologically (Davis, 1980). This functions as the self-reported methodology for measuring peoples’ interpretations of their ToM reasoning. It was found that there was a small-to-medium significant effect (*d* = 0.340) for this self-report measure, which indicates the value of self-report assessments, or at least the IRI as an assessment for ToM in adults. The social flexibility test is another self-report assessment that focuses on using specific selected items from the TEIQue assessment (TEIQue; Petrides, 2009). The items selected by Ikizer and Ramírez-Esparza to assess the construct focused on the ability to recognize how others and ourselves feel and how to deal with and manage those feelings (Ikizer & Ramírez-Esparza, 2018). Thus, this was an emotionally focused measure for assessing the affective dimension for ToM reasoning. Given that this self-report measure also found a significant difference with a close-to-medium effect size (*d* = 0.427), it would seem that self-report measures are likely a good direction to take in ToM assessments in adults. It can be argued that given that adults’ ToM reasoning is more advanced, more nuanced questions that can be found in self-report assessments are going to be better assessments compared to tasks that involve a simpler understanding of ToM.

The Reading in the Mind of the Eyes task (RMET) assesses mentalizing (Baron-Cohen et al., 2001), or identifying mental states based on images of eyes—another approach to studying ToM that found no significant difference in adults with a small effect size (Chung-Fat-Yim et al., 2022). This assessment takes both an emotional and social approach for understanding our ability to recognize the perspective of others’ emotions but may not be the direction that future studies should take for assessing ToM in adults, which may be due to ceiling effects. Finally, Cox and colleagues focused specifically on an older, aging population, and utilized a ToM measure that focuses on identifying whether a protagonist said something that is considered a “faux pas” in a series of short stories about social situations (Cox et al., 2016). This was also an assessment with a significant component of understanding social cues and norms. Although there was not a significant difference found (*p* = 0.060), the medium effect size found (*d* = 0.483) would indicate that this was still a robust study that would support more similar research to build on this finding. The specific focus on an aging population adds an additional dimension of understanding how aging interacts with language to play a role in our ToM reasoning capacity. This is especially the case given findings that indicate declining ToM in adulthood (Bernstein et al., 2011). More future studies can specifically approach the relationship of bilingualism with ToM in the context of aging populations.

Limitations of this systematic review include the small sample size and limited number of studies available. This means that any statistical analysis should be very carefully considered without definitive conclusions drawn. Potential biases by the reviewer during the screening process and limitations due to the terms included and excluded may have also affected the screening process. This includes the exclusion of key search terms like “additional language” or “foreign language” terms that may be used for bilingualism and may have excluded certain studies that used these terms instead of some of the other terms included. Future studies, especially once there are more studies added to the literature in this area of study, should include additional reviewers and perhaps more search terms, like “additional language” or “foreign language”.

The different dimensions illustrated through the different assessment tools utilized for understanding ToM demonstrate the range of interpretations of ToM. To better understand the differences that exist in adults in ToM reasoning, more studies are required to obtain a larger set of data for each of these different approaches. It is important to specifically study adults due to the nature of advanced ToM that is still developing in adults and because it is a more appropriate age to ask more complex questions regarding perspective-taking, including the understanding of more abstract aspects of others’ emotions. Utilizing these different tools may also help illuminate if there are differences in the effect of bilingualism depending on which aspect of ToM is assessed. It is useful to understand whether it is a cognitive, visual, emotional, social, or some other component of ToM reasoning that is most impacted by bilingualism, or whether there are consistent findings across these different dimensions. It is also valuable to understand ToM from behavioral, cognitive, and self-report data given the abstract nature of the concept of ToM, as this allows for diversified data for analyzing this relationship. Of course, it may also be valuable to develop new measures that focus on being able to detect ToM differences in adulthood.

Conducting more studies for adults will also help to uncover the role of language in ToM, especially with regard to a population that has a much more maturely developed language system. This is valuable for both a better understanding of bilingualism as well as a better understanding of ToM. For bilingualism, more studies with adults will better illuminate the distinction between the cognition of a bilingual adult and a monolingual adult. For ToM, more studies with adults will contribute to a better understanding of whether and in what areas there are differences in adults on ToM. Thus, we believe that it is important to expand the amount of research on the relationship of bilingualism and ToM for the population of adults to help strengthen this gap in the research.

## Figures and Tables

**Table 1:** Inclusion and exclusion criteria.

Inclusion Criteria	Exclusion Criteria
Compares some bilingual or multilingual group with a monolingual group Has some measure of theory of mind in some form that is behavioral or cognitive or self-report Includes a sample of neurotypical adults (18+)	Only includes a bilingual sample Uses neurological data

**Table 2:** Bilingualism and theory of mind in adult populations.

Study	ToM Measure	Monolingual Participants (N)	Bilingual Participants (N)	Average Age (Years)	Bilingual Advantage Found?	*p*-Value	Effect Size (*d*)
Rubio-Fernández and Glucksberg (2012)	Sally–Anne Task (eye-tracking)	23	23	Bilingual = 19.7 Monolingual = 19.4	Yes	<0.045	0.624
Tarighat and Krott (2021)	Self-Reported Perspective-Taking	108	108	Bilingual = 33.045 Monolingual = 33.015	Yes	0.012	0.340
Navarro and Conway (2021)	Director Task	26	28	Bilingual = 27.29 Monolingual = 25.48	Yes	0.027	0.510
Chung-Fat-Yim et al. (2022)	Reading in the Mind of the Eyes Task	840	1155	Bilingual = 20.4 Monolingual = 27.4	Yes	<0.001	−0.160
Ryskin et al. (2014)	Visuospatial	31	33	20.07	No (more errors by bilinguals)	<0.05	0.168
Ikizer and Ramírez-Esparza (2018)	Social Flexibility	465	206	Bilingual = 37 Monolingual = 41.12	Yes	<0.001	0.427
Cox et al. (2016)	Faux Pas Test	64	26	Bilingual = 74.54 Monolingual = 74.45	No	0.060	0.482

## Data Availability

No new data were created or analyzed in this study.
