# Correction: Xia and Haas (2025). A Systematic Review on the Association Between Bilingualism and Theory of Mind in Adulthood. *Behavioral Sciences, 15*(6), 815

**DOI:** 10.3390/bs15101422

**Published:** 2025-10-20

**Authors:** Rowena J. Xia, Brian W. Haas

**Affiliations:** 1 Department of Psychology, Concordia College, Moorhead, MN 56562, USA; 2 Department of Psychology, University of Georgia, Athens, GA 30602, USA; bhaas@uga.edu

There was an error in the original publication (Xia & Haas, 2025). An incorrect effect size was included due to incorrect data used for one of the reviewed articles (Chung-Fat-Yim et al., 2022).

A correction has been made to Abstract:

Previous research on the relationship of bilingualism and theory of mind has largely focused on children. However, several recent studies of the theory of mind have found differences in theory of mind processing among older populations, namely adults. Given that language has been found to play an important role in the successful theory of the mind task performance of adults, it is valuable to understand the relationship of the language ability of bilingualism and theory of mind in adults. The specific focus is on studies comparing monolinguals and bilinguals in a theory of mind assessment for an adult sample. In this systematic review, we reviewed and analyzed these studies and conducted a meta-analysis. Among the studies included for meta-analysis (k = 7), we found a significant small-to-medium effect size (*d* = 0.402, *p* < 0.0001), indicating a bilingual advantage among adults. A variety of different measures for theory of mind were included in these studies. More studies are required to better understand the relationship between multiple language processing and social cognition among adults to better understand this gap in the literature.

There was an error in the original publication. Some of the statistical analysis was inaccurate due to incorrect data used for one of the reviewed articles (Chung-Fat-Tun et al., 2022). The corrected statistics have been included.

A correction has been made to Results, Paragraph 2:

A funnel plot (Figure 2) was generated to analyze potential publication bias. A visual inspection of the funnel plot does not appear to demonstrate asymmetry, indicating a lack of publication bias. Using the random-effects model analysis, the estimated effect was found to be *d* = 0.402, *p* < 0.0001, 95% CI [0.2901, 0.5143], indicating a significant medium effect size. The results indicate a bilingual advantage found in adults. The tests of heterogeneity for the model indicate that it is unlikely that there is heterogeneity among the studies with τ^2^ = 0, CI [0.0000, 0.2727], and *Ι*^2^ = 20.2%. Cochran’s Q test statistics indicate a *p*-value of 0.276, which indicates a non-significant test of heterogeneity for this model. Given the small number of studies included (N = 7), it is with caution that these tests of heterogeneity are interpreted, especially given that every study included a different type of ToM measure. The sensitivity and robustness of this meta-analysis is heavily restricted by the sample size (N = 7). The forest plot for the studies included in this meta-analysis can be found in Figure 3.

In the original publication, there was a mistake in Table 2 as published. The participant numbers (N) were incorrect from both monolingual and bilingual participants based on errors reported by the authors. The effect size value and *p*-value was also incorrect and has now been corrected along with the response for the “Bilingual Advantage Found” column. The corrected Table 2 appears below. 

In the original publication, there was a mistake in Figure 2 as published. The funnel plot was incorrect due to incorrect data provided for one study (Chung-Fat-Yim et al., 2022). The corrected Figure 2 appears below. 

**Figure 2:**
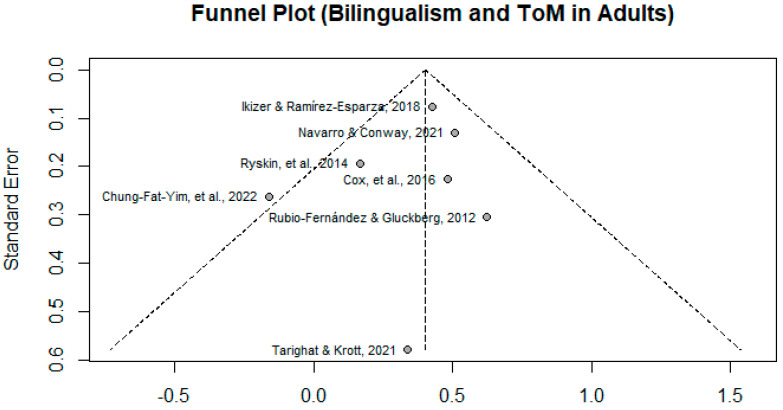
Funnel plot for meta-analysis of studies. (Ikizer & Ramírez-Esparza, 2018; Navarro & Conway, 2021; Ryskin et al., 2014; Cox et al., 2016; Rubio-Fernández & Glucksberg, 2012; Tarighat & Krott, 2021; Chung-Fat-Yim et al., 2022).

In the original publication, there was a mistake in Figure 3 as published. The original forest plot made was inaccurate due to incorrect data for one study (Chung-Fat-Yim et al., 2022). The corrected Figure 3 appears below. 

**Figure 3:**
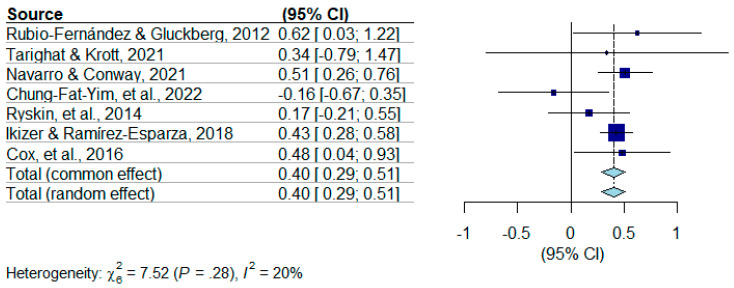
Forest plot for meta-analysis of studies. (Chung-Fat-Yim et al., 2022; Cox et al., 2016; Ikizer & Ramírez-Esparza, 2018; Navarro & Conway, 2021; Rubio-Fernández & Glucksberg, 2012; Ryskin et al., 2014; Tarighat & Krott, 2021).

There was an error in the original publication. The total random effect stated in the discussion was calculated based on previous incorrect data for one of the studies and has now been corrected with updated total random effect.

A correction has been made to Discussion, Paragraph 2:

As can be seen through the forest plot, the majority of the seven studies have a 95% confidence interval indicating a significant effect (Figure 3). Several studies have an effect size settling close to the total random effect, *d* = 0.402. Additionally, the test of heterogeneity of these studies indicates that the inconsistency is very low, *I*^2^, which supports a tentatively confident interpretation of this meta-analytic result as robust, with the caveat of the limited sample size (n = 7). 

In the original publication, the Data Availability Statement was missing. The data availability statement was updated to indicate that no new data was created or analyzed for this study.

The correction has been made as follows:

Data Availability Statement: No new data were created or analyzed in this study. 

The authors state that the scientific conclusions are unaffected. This correction was approved by the Academic Editor. The original publication has also been updated.

## Figures and Tables

**Table 2:** Bilingualism and theory of mind in adult populations.

Study	ToM Measure	Monolingual Participants (N)	Bilingual Participants (N)	Average Age (Years)	Bilingual Advantage Found?	*p*-Value	Effect Size (*d*)
Rubio-Fernández and Glucksberg (2012)	Sally–Anne Task (eye-tracking)	23	23	Bilingual = 19.7 Monolingual = 19.4	Yes	<0.045	0.624
Tarighat and Krott (2021)	Self-Reported Perspective-Taking	108	108	Bilingual = 33.045 Monolingual = 33.015	Yes	0.012	0.340
Navarro and Conway (2021)	Director Task	26	28	Bilingual = 27.29 Monolingual = 25.48	Yes	0.027	0.510
Chung-Fat-Yim et al. (2022)	Reading in the Mind of the Eyes Task	840	1155	Bilingual = 20.4 Monolingual = 27.4	Yes	<0.001	−0.160
Ryskin et al. (2014)	Visuospatial	31	33	20.07	No (more errors by bilinguals)	<0.05	0.168
Ikizer and Ramírez-Esparza (2018)	Social Flexibility	465	206	Bilingual = 37 Monolingual = 41.12	Yes	<0.001	0.427
Cox et al. (2016)	Faux Pas Test	64	26	Bilingual = 74.54 Monolingual = 74.45	No	0.060	0.482
